# Supplementary material for: Biofilm formation displays intrinsic offensive and defensive features of Bacillus cereus
Source: NPJ Biofilms Microbiomes. 2020 Jan 15;6:3. doi: 10.1038/s41522-019-0112-7 (PMC6962202; doi:10.1038/s41522-019-0112-7)
Supplement: Supplementary file 1 — Supplementary material [file 41522_2019_112_MOESM1_ESM.pdf]

Biofilm formation displays intrinsic offensive and defensive features of *Bacillus cereus*

Caro-Astorga Joaquin[1], Frenzel Elrike[2], Perkins James R[3], Álvarez-Mena Ana[1], de Vicente Antonio[1], Ranea Juan A.G.[4], Kuipers Oscar P.[2], and Romero Diego[1]*

Supplementary information

Supplementary Figures

Supplementary Tables

Supplementary data 1. Manual annotated results.xls

Supplementary movies:

Supplementary movie_1-Biofilm + HeLa Conc 4 - Well F3.avi

Supplementary movie_2-Planktonic +HeLa Conc 4 - Well F9.avi

Supplementary movie_3-Biofilm +MDA Conc 2 - Well B2 timelapse.avi

Supplementary movie_4-Planktonic +MDA Conc 2 - Well B8.avi

Supplementary Figure 1

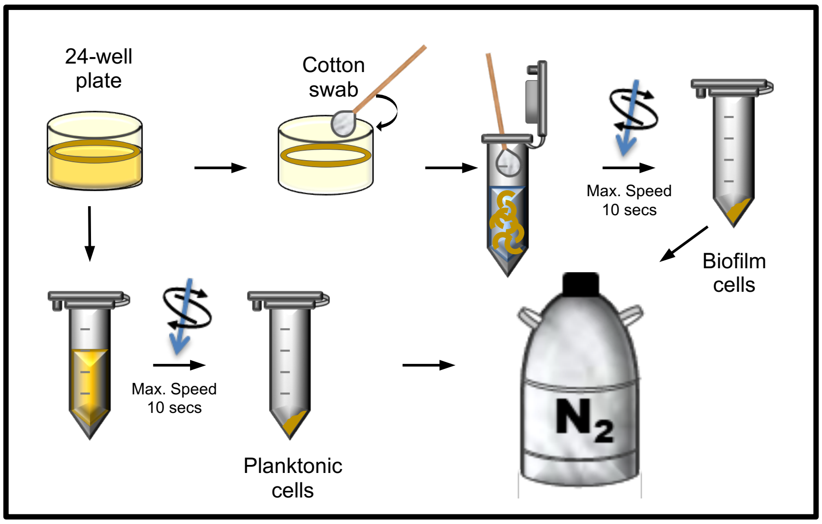

Supplementary Figure 1. Isolation method of biofilm and floating cells. Biofilms were grown in 24-well plates without agitation at 28ºC. Floating cells were harvested taking only 250-500 µl of the upper part of the medium without disturbing the submerged biofilm. Samples were immediately centrifuged at maximum speed for 10 seconds and frozen in liquid nitrogen after discarding the supernatant. Next, the remaining medium and submerged biofilm was discarded, and the biofilm was collected with sterile cotton swabs, suspended in 1 ml of TY medium, centrifuged at maximum speed for 10 seconds and frozen in liquid nitrogen after discarding the supernatant. The sampling time was less than one minute to prevent RNA degradation and RNA recycling.

Supplementary Figure 2

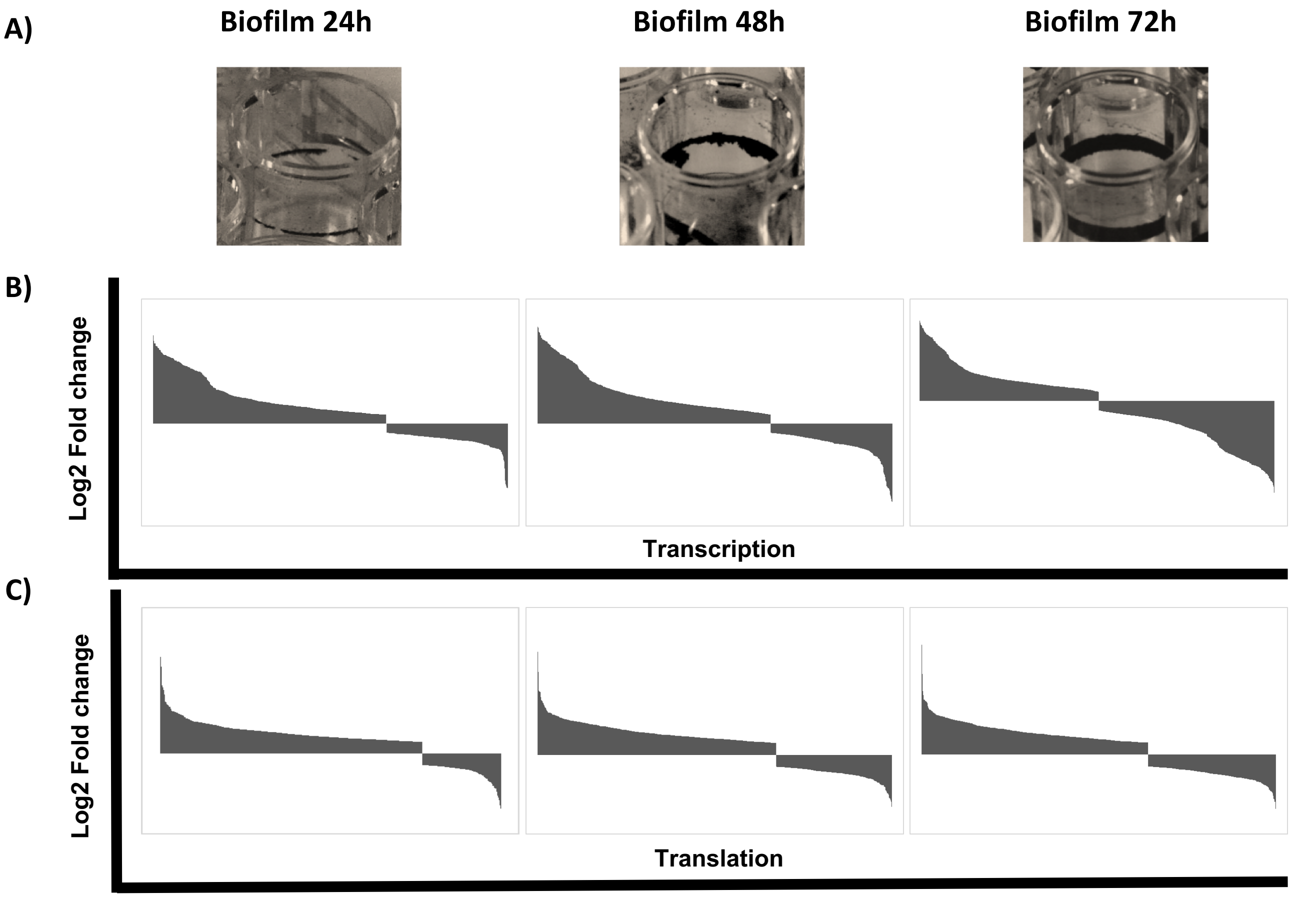

Supplementary Figure 2. A) Pictures of biofilm of *B. cereus* at 24, 48 and 72 h, stained with crystal violet and washed several time in water. B) Graph Log_2 Fold Change of genes transcription levels at 24, 48 and 72 h. Statistically significant changes are shown ($Log_2 > 2$ and p-value < 0.05). C) Graph Log_2 (Fold Change) of protein levels at 24, 48 and 72 h. Statistically significant changes are shown ($Log_2 > 0.7$ and q-value < 0.05).

Supplementary Figure 3

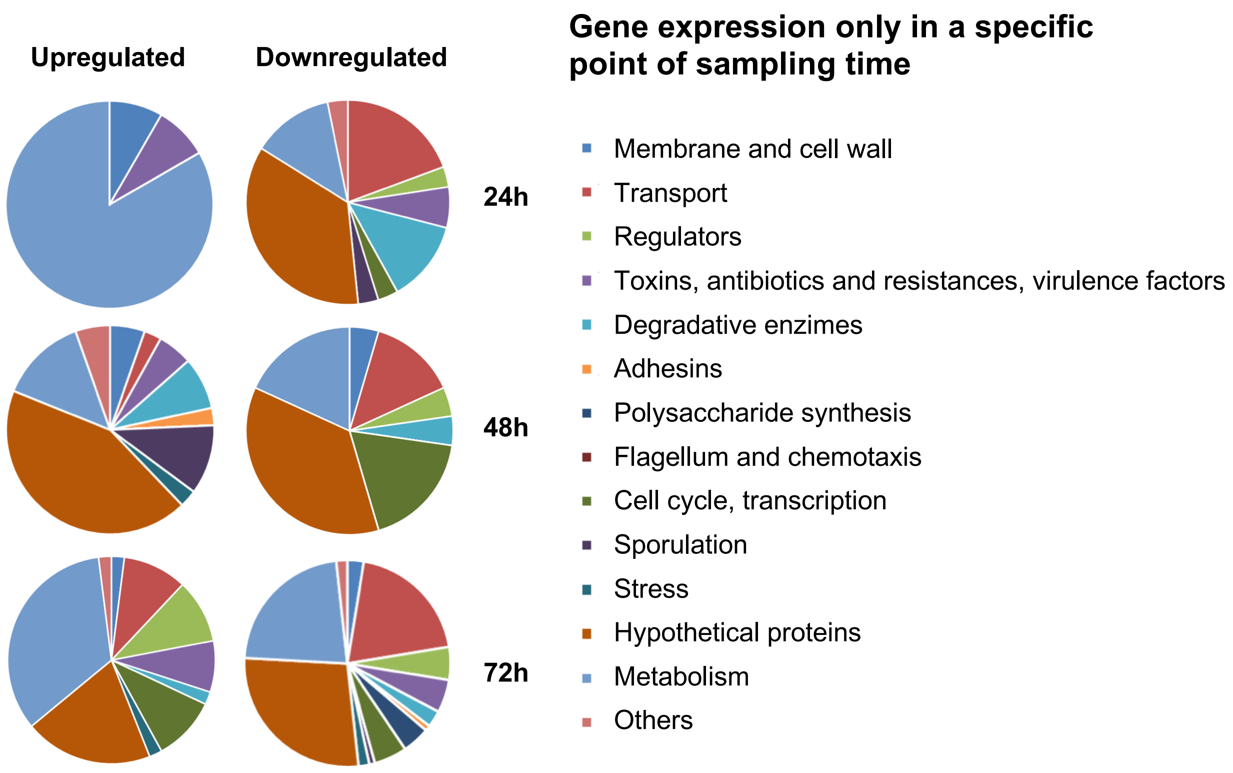

Supplementary Figure 3. Circle charts of genes with statistically significant changes in their expression pattern (Log2 (Fold change) > |2|) at 24, 48 or 72 h but not at other time points. Specific stage genes increase their diversity especially at 48 and 72 h biofilms.

Supplementary Figure 4

A)

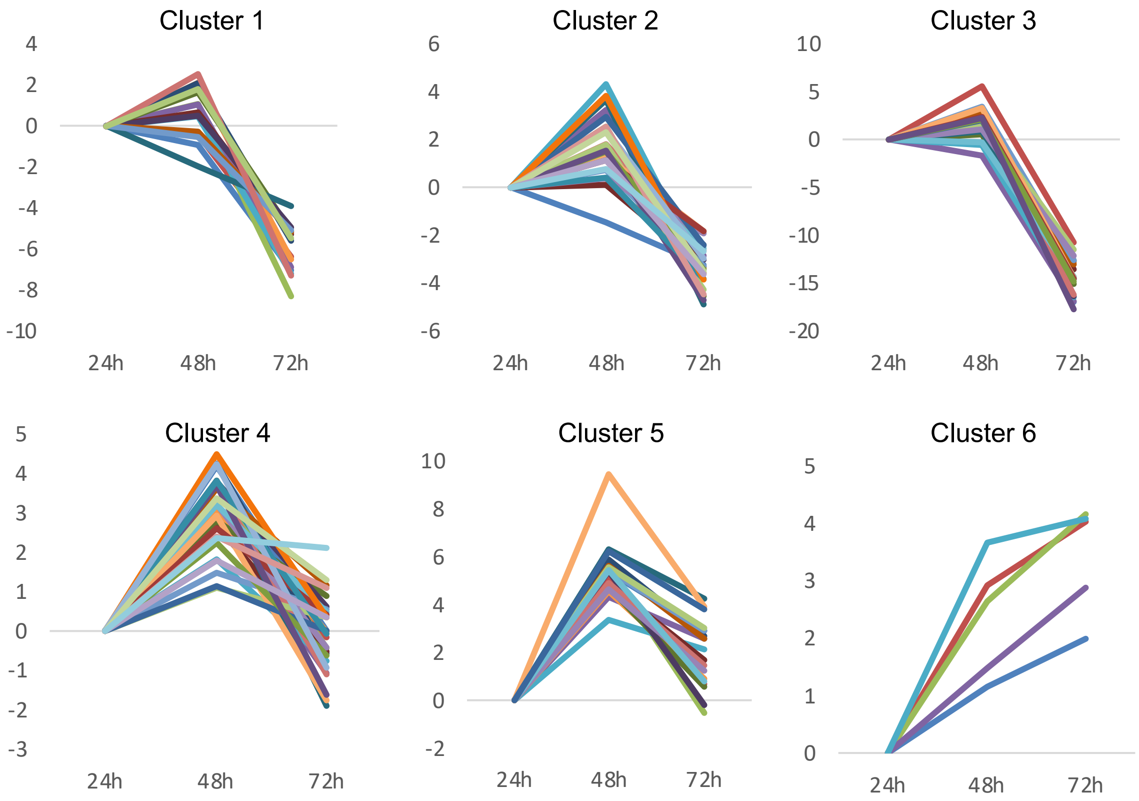

B) **Cluster 6**

Gene ID	24h	48h	72h	Gen name
BC2353	1.47	2.61	3.46	Spo0A-P phosphatase
BC2142	-0.32	2.59	3.71	stage V sporulation protein S
BC1340	-0.45	2.19	3.71	Sporulation kinase KinE
BC2259	3.21	3.81	3.80	Sporulation-control protein Spo0M
BC5482	-3.31	0.36	0.77	Sporulation initiation inhibitor protein soj

Supplementary Figure 4. A) Clustering of sporulation genes with STEM. Clusters 1-5 show sharp downregulation of most of sporulation genes. Cluster 6 contain five genes with an expression pattern of higher expression at 72 h than in previous strages. B) List of genes included in cluster 6 showing that three out of five genes are involved in negative regulation of sporulation and one gene involved in the last stage of spore maturation, proposed to play a role in the coat assembly. KinE, has been demonstrated low phosphorylation activity to the sporulation phosphorelay (Fujita and Losick, 2005).

Supplementary Figure 5

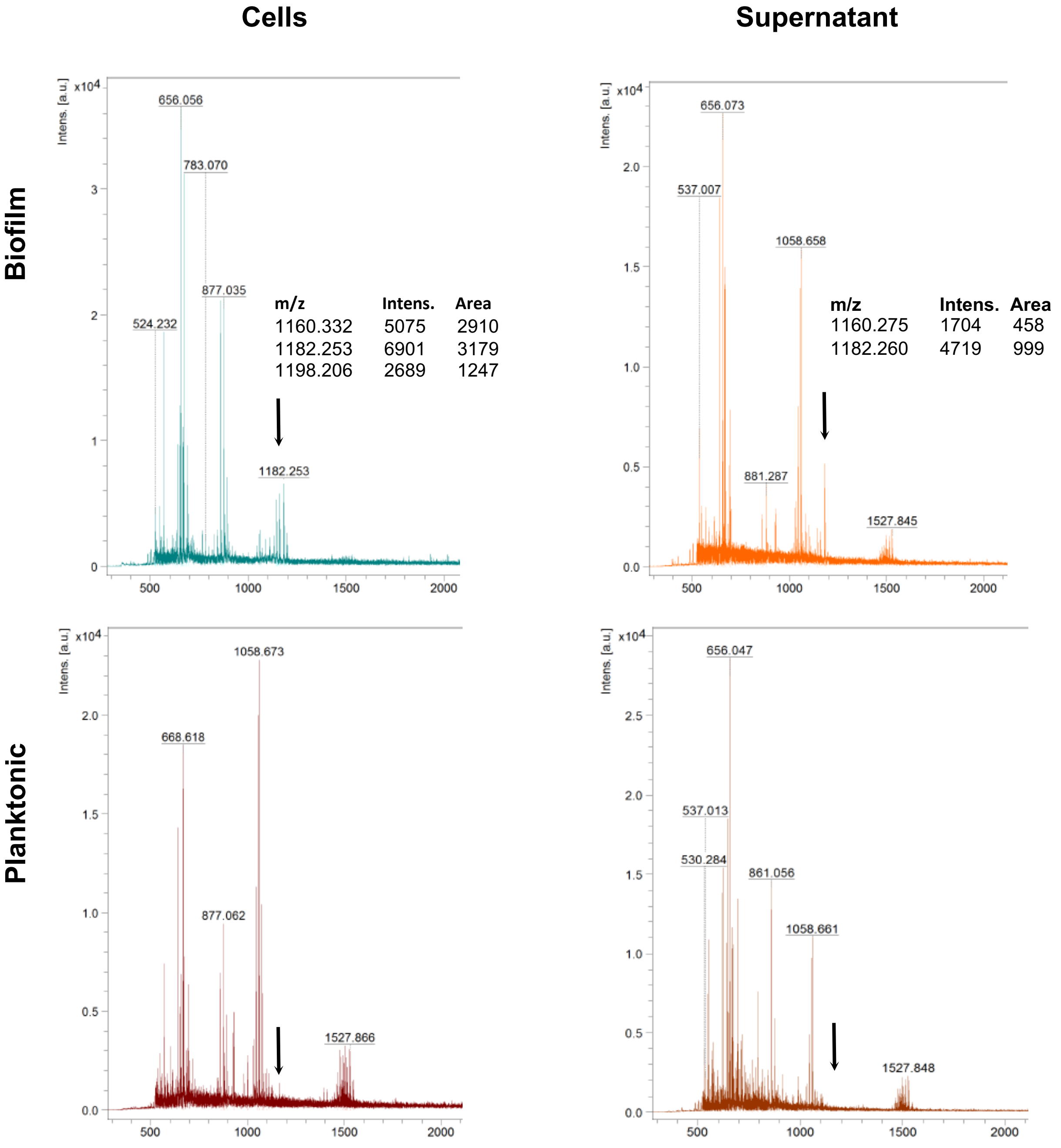

Supplementary Figure 5. Complete spectrums from Mass-spectrometry HPLC-MS-MS (Tof-Tof) of cells and supernatants of 48 h cultures. Biofilm was collected and suspended in PBS, separating cells from supernatant with centrifugation. Culture medium was centrifuged to separate floating cells from supernatant. Samples were purified with C8 ZipTip® (Merk) previous to MS analysis. Tables resume the m/z found corresponding to Thiocillin (m/z=1160) and sodium (m/z=1182) and potassium adducts (m/z=1198). Black arrows indicate approximate positions of Thiocillin peaks.

Supplementary Figure 6

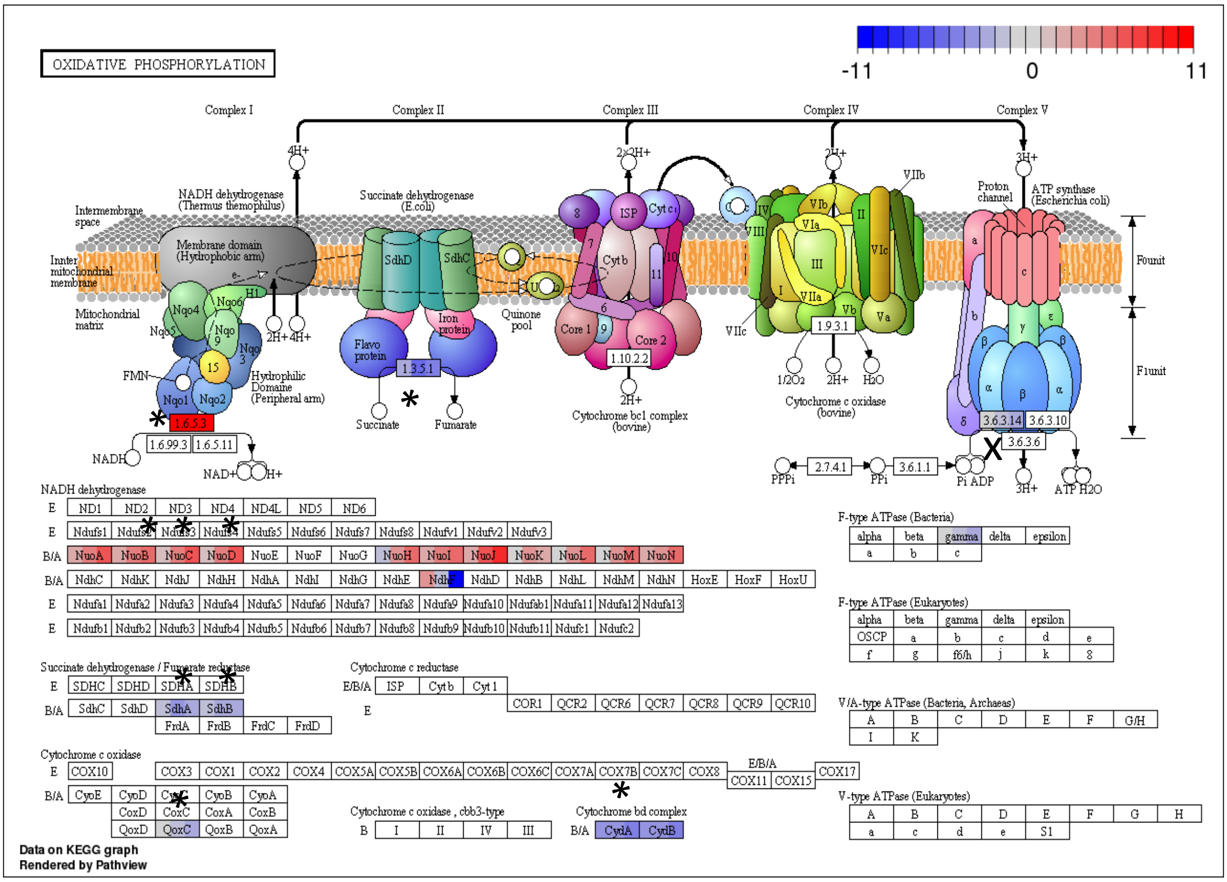

Supplementary Figure 6. Oxidative phosphorylation pathway scheme showing the overexpression of the Complex I in biofilm cells and downregulation of complex II. Asterisk (*) indicates confirmation of the behavior by iTRAQ results and cross (X) indicates discordant result with proteomic results.

Supplementary Figure 7

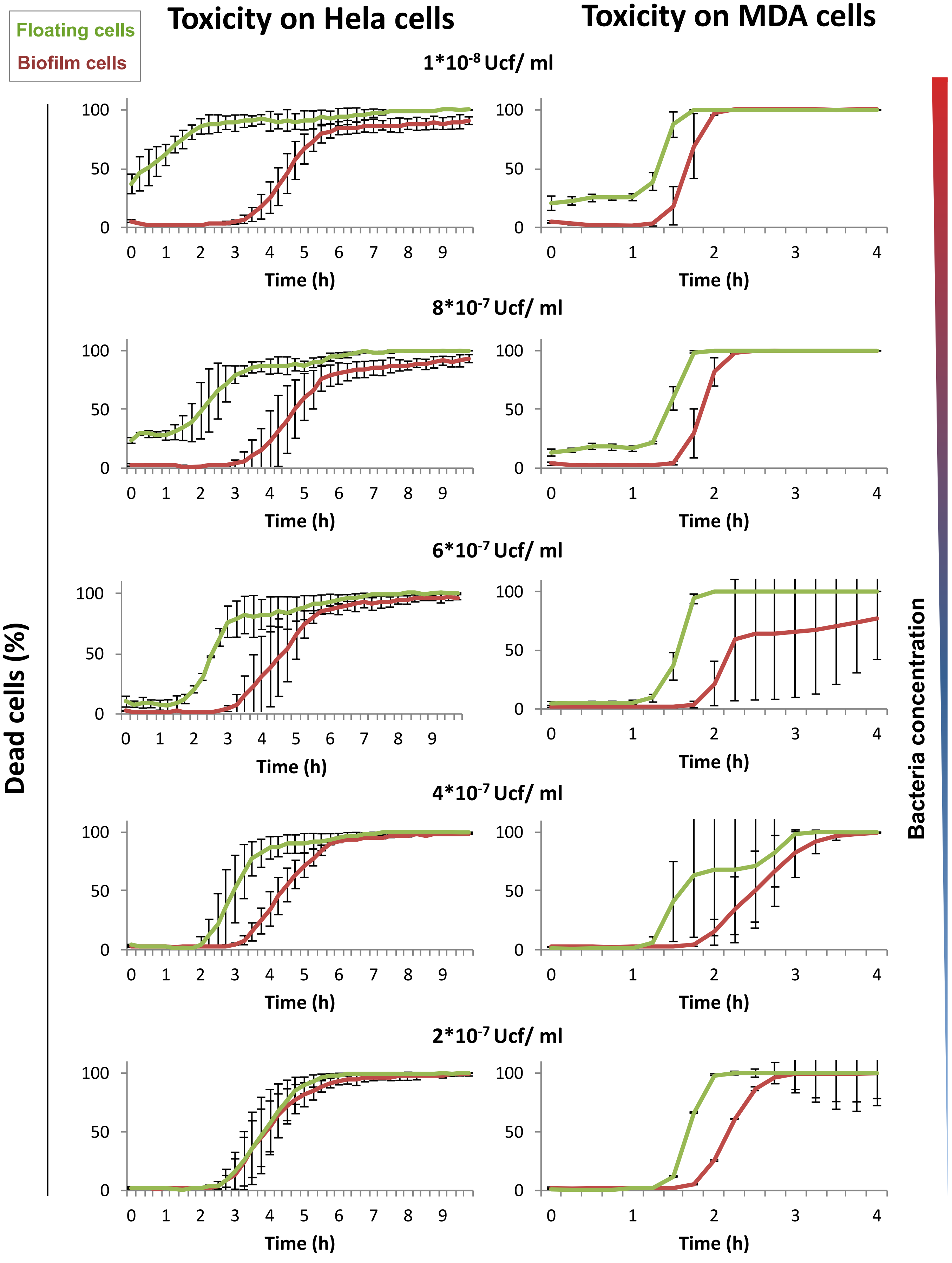

Supplementary Figure 7. Toxicity assay of floating cells (green line) or biofilm (red line) cells of 48 h bacterial cultures against HeLa and MDA cell lines cultures without antibiotics. Error bars indicate SD (n=12). Bacteria samples were washed in PBS twice and the OD600 was adjusted to 1 (~10^7 ucf/ml). From this suspension, serial dilutions were done previous to inoculation.

Supplementary Figure 8

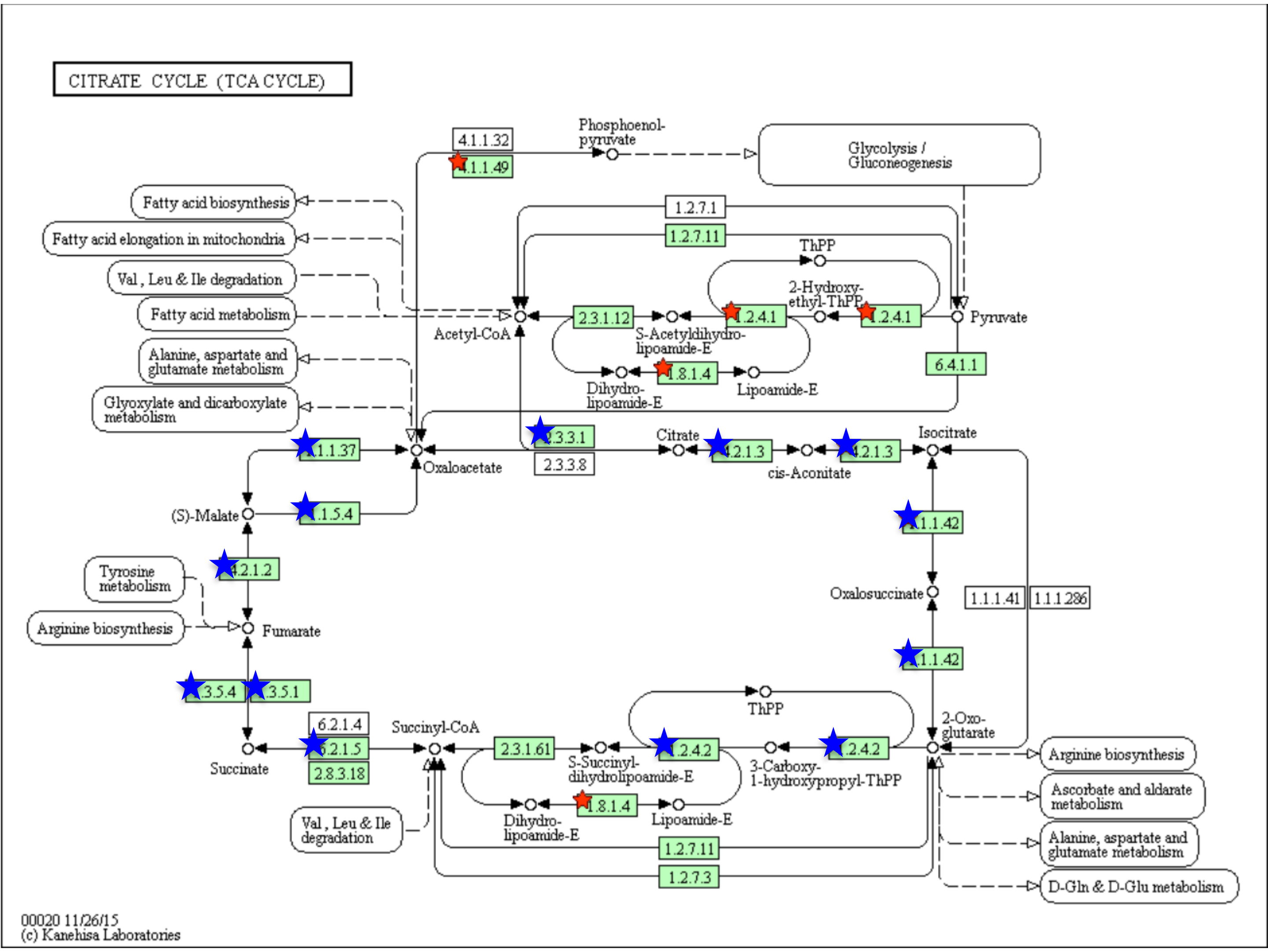

Supplementary Figure 8. Canonic pathway of Tricarboxilic Acid Cycle (TCA) (KEGG Database). Squares indicate enzymatic processes. Green squares indicate process able to be accomplished by *B. cereus*. Stars indicate enhanced expression in planktonic cells (blue) or biofilm cells (red).

Supplementary Figure 9

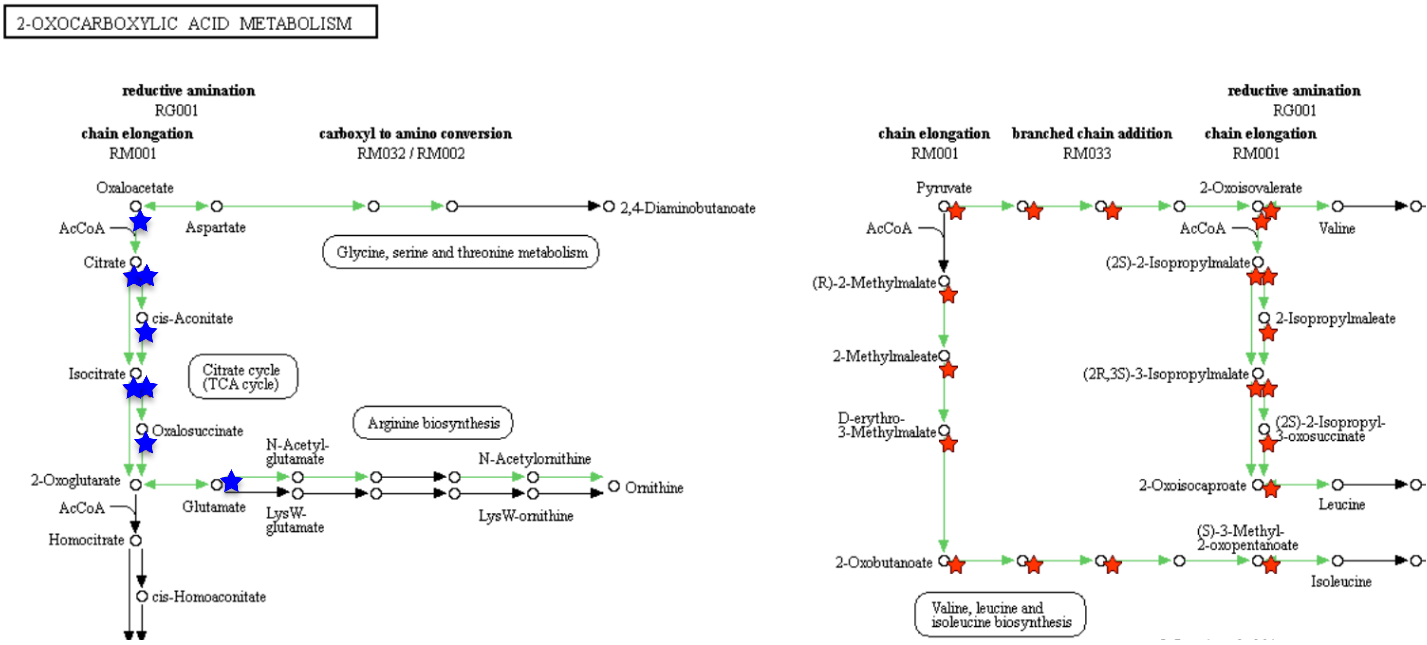

Supplementary Figure 9. Canonic scheme of the 2-Oxocarboxylic Acid metabolism (KEGG Database). Arrows indicate enzymatic processes. Green arrows indicate process able to be accomplished by *B. cereus*. Stars indicate enhanced expression in planktonic cells (blue) or biofilm cells (red).

Supplementary Figure 10

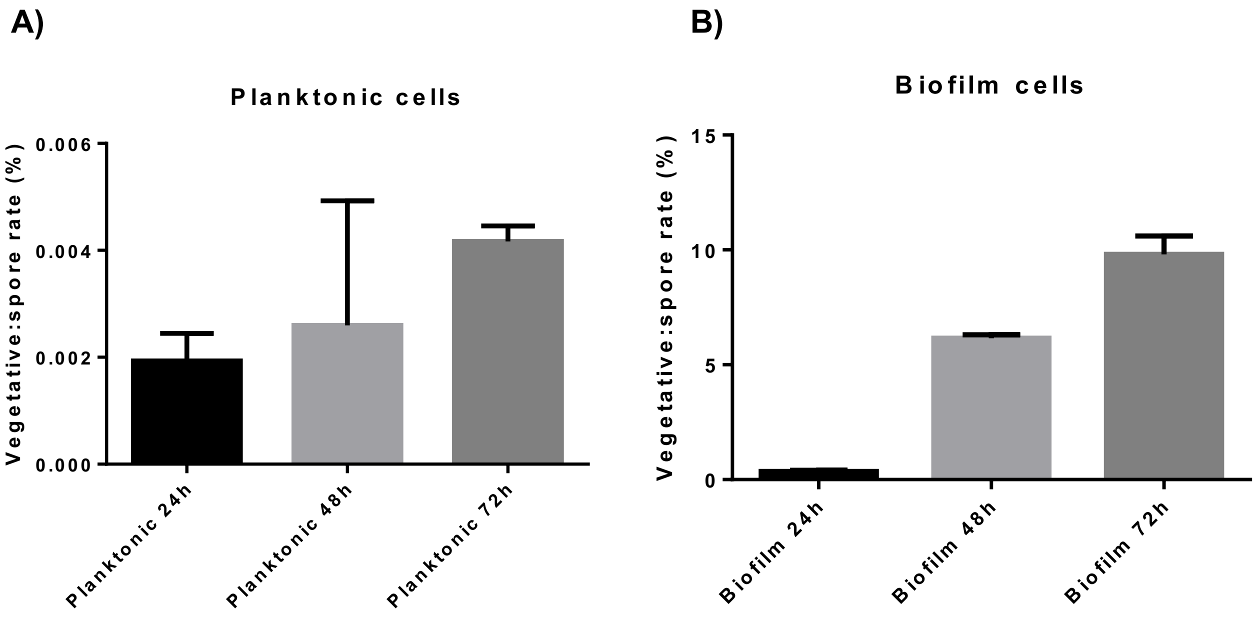

Supplementary Figure 10. Percentage of sporulation in planktonic (floating) or biofilm associated cells at different time points. Values are the mean of three replicates and standard deviation.

Supplementary Tables

Supplementary Table 1. Expression pattern of genes involved in amino acid transport. Asterisk indicates confirmed behaviour at protein levels.

		Log2 (fold change)			
**Gene ID**	**24 h**	**48 h**	**72 h**	**Gene name**	**iTRAQ**
BC0401	1.38	4.05	4.71	cystine transport system permease	*
BC0402	2.46	4.30	5.42	cystine-binding protein	*
BC0403	1.24	4.12	4.70	glutamine transport ATP-binding protein glnQ	*
BC0638	1.44	4.50	5.10	Sodium/proton-dependent alanine carrier protein	*
BC0639	8.70	8.82	1.78	glutamine transport ATP-binding protein glnQ	
BC0640	15.73	17.36	7.72	glutamine-binding protein	*
BC0703	1.23	3.41	3.47	Sodium/proline symporter	
BC0865	0.02	-0.51	-3.69	arginine/ornithine antiporter	
BC1231	-3.21	-1.45	-0.07	Sodium/proline symporter	
BC1432	-1.59	-1.90	-3.18	proton/sodium-glutamate symport protein	*
BC1609	1.55	4.17	5.94	Sodium/proline symporter	*
BC1927	2.11	5.79	6.66	leu-, iso-, val-, trn-, ala-binding protein	*
BC2790	5.31	7.63	5.29	glycine betaine transport system permease	
BC2980	-0.14	-0.53	-4.31	arginine permease	
BC3398	0.01	-2.47	-5.75	Serine transporter	*
BC4071	2.82	5.62	6.82	Sodium/proline symporter	*
BC4149	-2.26	-3.48	-1.42	arginine ABC transporter permease	*
BC4150	-2.13	-2.90	-0.30	arginine-binding protein	*
BC4242	1.12	2.20	4.00	proton/sodium-glutamate symport protein	
BC5043	-1.63	-3.20	-4.10	Sodium/proton-dependent alanine carrier protein	
BC5051	-3.53	-1.60	-3.76	Sodium/proton-dependent alanine carrier protein	
BC5218	-0.03	-3.89	-16.49	proton/sodium-glutamate symport protein	

Supplementary Table 2. Expression pattern of genes involved in antimicrobial biosynthesis. Asterisk indicates confirmed behaviour at protein levels.

	Log2 (fold change)				
**Gene ID**	**24 h**	**48 h**	**72 h**	**Molecule**	**iTRAQ**
BC1201	2.87	5.63	1.75	Tylosin	
BC1204	8.07	9.53	5.09	Tylosin	
BC1206	7.01	8.13	2.73	Tylosin	*
BC1208	5.00	6.74	1.04	Streptomycin like	
BC1209	5.95	7.91	2.46	Streptomycin like	*
BC1210	3.47	4.24	-0.52	Streptomycin like	
BC1211	3.78	6.19	2.59	Streptomycin like	
BC1212	4.36	6.94	0.28	Streptomycin like	*
BC1213	3.38	5.85	-1.63	Streptomycin like	
BC1214	3.10	5.17	0.92	Streptomycin like	*
BC1248	3.77	5.88	2.80	Bacteriocin	
BC1249	4.28	5.43	3.24	Bacteriocin	
BC1250	4.31	5.00	1.78	Bacteriocin	
BC2452	0.24	3.00	4.99	Bacitracin	
BC2966	16.71	17.72	10.24	Polyketide synthase	
BC5079	-1.90	3.60	4.62	Thiocillin	*
BC5080	-1.58	3.00	3.75	Thiocillin	*
BC5081	-0.86	3.27	4.33	Thiocillin	*
BC5082	-2.43	3.20	4.32	Thiocillin	*
BC5083	-2.33	2.06	3.34	Thiocillin	*
BC5084	-1.76	2.48	3.69	Thiocillin	*
BC5085	-0.77	3.52	3.36	Thiocillin	*
BC5086	-1.76	2.83	4.04	Thiocillin	*
BC5087	2.85	3.61	3.02	Thiocillin	
BC5088	2.81	3.74	3.14	Thiocillin	
BC5089	2.61	3.65	2.90	Thiocillin	
BC5090	2.61	2.87	3.13	Thiocillin	
BC3021	0.48	2.21	2.78	Colicin	*
BC1426	2.01	8.10	8.50	Porphirin	
BC1427	1.46	5.19	4.77	Porphirin	
BC1428	3.04	6.66	6.96	Porphirin	*
BC2133	-2.57	-4.22	-11.87	Porphirin	
BC2134	-2.99	-4.05	-6.25	Porphirin	
BC4468	2.84	3.27	3.62	Porphirin	

Supplementary Table 3. Specialization of biofilm and floating cells in distinct but coordinated metabolic activities. Genes belonging to cluster 1.

GeneIDs	Planktonic(blue)/Biofilm(red)	DAVID Functional annotation
BC0344	blue	1-pyrroline-5-carboxylate dehydrogenase
BC0410	blue	Crp family transcriptional regulator
BC0411	blue	hypothetical protein
BC0466	blue	fumarate hydratase
BC0491	blue	formate acetyltransferase
BC0492	blue	pyruvate formate-lyase activating enzyme
BC0589	blue	formate dehydrogenase alpha chain
BC0590	blue	hypothetical protein
BC0611	blue	aspartate ammonia-lyase
BC0612	blue	L-lactate permease
BC0621	blue	2-amino-3-ketobutyrate CoA ligase
BC0849	blue	acetyltransferase
BC0898	blue	enoyl-CoA hydratase
BC1149	blue	ornithine--oxo-acid transaminase
BC1231	blue	sodium/proline symporter
BC1252	blue	2-oxoglutarate dehydrogenase subunit E1
BC1746	blue	asparagine synthetase AsnA
BC2220	blue	alcohol dehydrogenase
BC2758	blue	metal-dependent hydrolase
BC2896	blue	aspartate aminotransferase
BC2959	blue	malate:quinone oxidoreductase
BC3616	blue	aconitate hydratase
BC3650	blue	imidazolonepropionase
BC3651	blue	urocanate hydratase
BC3652	blue	histidine ammonia-lyase
BC3653	blue	anti-terminator HutP
BC3833	blue	succinyl-CoA synthetase subunit alpha
BC3834	blue	succinyl-CoA synthetase subunit beta
BC4023	blue	acetyl-CoA acetyltransferase
BC4224	blue	glycine dehydrogenase subunit 2
BC4225	blue	glycine dehydrogenase subunit 1
BC4226	blue	glycine cleavage system aminomethyltransferase T
BC4365	blue	bifunctional acetaldehyde-CoA/alcohol dehydrogenase
BC4516	blue	succinate dehydrogenase iron-sulfur subunit
BC4517	blue	succinate dehydrogenase flavoprotein subunit
BC4592	blue	malate dehydrogenase
BC4593	blue	isocitrate dehydrogenase
BC4594	blue	citrate synthase
BC4870	blue	L-lactate dehydrogenase

BC4995	blue	regulatory protein
BC4996	blue	L-lactate dehydrogenase
BC5002	blue	acyl-CoA dehydrogenase
BC5003	blue	acetyl-CoA acetyltransferase
BC5004	blue	enoyl-CoA hydratase
BC5006	blue	Prolyne dehydrogenase
BC5228	blue	L-lactate permease
BC5342	blue	acyl-CoA dehydrogenase
BC5344	blue	acetyl-CoA acetyltransferase
BC5345	blue	Iron-sulphur-binding reductase
BC5438	blue	antiholin-like protein LrgB
BC5439	blue	murein hydrolase regulator LrgA
BC0355	red	4-aminobutyrate--2-oxoglutarate transaminase
BC0356	red	sigma-54-dependent transcriptional activator
BC0357	red	succinate-semialdehyde dehydrogenase [NADP+]
BC1036	red	glycerol-3-phosphate dehydrogenase
BC1301	red	two component system histidine kinase
BC1396	red	branched-chain amino acid aminotransferase
BC1398	red	acetolactate synthase small subunit
BC1399	red	ketol-acid reductoisomerase
BC1400	red	2-isopropylmalate synthase
BC1401	red	3-isopropylmalate dehydrogenase
BC1402	red	3-isopropylmalate dehydratase large subunit
BC1610	red	aminotransferase
BC1611	red	hypothetical protein
BC1776	red	branched-chain amino acid aminotransferase
BC1777	red	acetolactate synthase 3 catalytic subunit
BC2285	red	citrate synthase 3
BC2286	red	2-methylcitrate dehydratase
BC2287	red	methylisocitrate lyase
BC2288	red	acyl-CoA dehydrogenase
BC2289	red	3-hydroxyisobutyrate dehydrogenase
BC2290	red	methylmalonate-semialdehyde dehydrogenase
BC2292	red	3-hydroxyisobutyryl-CoA hydrolase
BC2776	red	dihydrolipoamide dehydrogenase
BC2778	red	acetoin dehydrogenase E1 component beta-subunit
BC3555	red	aldehyde dehydrogenase
BC3939	red	formamidase
BC4325	red	late competence protein ComER
BC4595	red	hypothetical protein
BC4644	red	PhnB protein
BC4645	red	acetyl-coenzyme A synthetase
BC4762	red	phosphoenolpyruvate carboxykinase
BC5133	red	holin-like protein

Supplementary Table 4. KEGG pathways enrichment data for ALL, blue (planktonic), and red (biofilm) genes in cluster 1. Significance tests p-value and Benjamini are shown, and the number and percentage of gene over the total genes in Cluster 1 is also shown for each KEGG pathway.

KEGG: Metabolic pathways	P-Value	Benjamini	#genes	%genes
ALL	8.00E-10	6.80E-09	52	62.7
BLUE (Planktonic)	3.50E-07	2.10E-06	34	66.7
RED (Biofilm)	1.70E-03	6.10E-03	18	56.2

KEGG: Biosynthesis of antibiotics	P-Value	Benjamini	#genes	%genes
ALL	5.80E-14	2.90E-12	35	42.2
BLUE (Planktonic)	1.60E-10	6.60E-09	24	47.1
RED (Biofilm)	5.00E-04	2.30E-03	11	34.4

KEGG: Biosynthesis of secondary metabolites	P-Value	Benjamini	#genes	%genes
ALL	8.90E-13	2.30E-11	40	48.2
BLUE (Planktonic)	7.10E-08	5.90E-07	25	49
RED (Biofilm)	1.90E-05	1.70E-04	15	46.9

KEGG: Citrate cycle (TCA cycle)	P-Value	Benjamini	#genes	%genes
ALL	9.00E-13	1.50E-11	15	18.1
BLUE (Planktonic)	1.00E-09	1.40E-08	11	21.6
RED (Biofilm)	8.70E-03	2.10E-02	4	12.5

KEGG: 2-Oxocarboxylic acid metabolism	P-Value	Benjamini	#genes	%genes
ALL	2.80E-10	2.80E-09	13	15.7
BLUE (Planktonic)	4.00E-02	1.00E-01	4	7.8
RED (Biofilm)	1.70E-09	3.20E-08	9	28.1

KEGG: Glyoxylate and dicarboxylate metabolism	P-Value	Benjamini	#genes	%genes

ALL	4.10E-09	2.60E-08	13	15.7
BLUE (Planktonic)	1.10E-07	7.60E-07	10	19.6
RED (Biofilm)	9.30E-02	1.90E-01	3	9.4

**KEGG: Pyruvate metabolism**	**P-Value**	**Benjamini**	**#genes**	**%genes**
ALL	2.20E-08	1.20E-07	15	18.1
BLUE (Planktonic)	7.50E-05	3.40E-04	9	17.6
RED (Biofilm)	1.10E-03	4.40E-03	6	18.8

**KEGG: Propanoate metabolism**	**P-Value**	**Benjamini**	**#genes**	**%genes**
ALL	5.50E-08	2.80E-07	11	13.3
BLUE (Planktonic)	5.70E-05	2.90E-04	7	13.7
RED (Biofilm)	7.10E-03	2.00E-02	4	12.5

**KEGG: Butanoate metabolism**	**P-Value**	**Benjamini**	**#genes**	**%genes**
ALL	1.20E-07	5.20E-07	11	13.3
BLUE (Planktonic)	8.90E-05	3.60E-04	7	13.7
RED (Biofilm)	8.70E-03	2.10E-02	4	12.5

Supplementary Table 5. Gene composition of three general enriched KEGG pathways in Cluster 1 is indicated by "x" symbol.

KEGG: Metabolic pathways	KEGG: synthesis of secondary metabolites	KEGG: synthesis of antibiotics	Functional annotation	Gene
x			1-Pyrroline-5-Carboxylate dehydrogenase	BC0344
x			4-Aminobutyrate--2-Oxoglutarate transaminase	BC0355
	x		Glycerol-3-Phosphate dehydrogenase	BC1036
x			Succinate-Semialdehyde dehydrogenase [NADP+]	BC0357
x	x	x	Fumarate hydratase	BC0466
x			Formate acetyltransferase	BC0491
x			formate dehydrogenasealpha chain	BC0589
x			aspartate ammonia-lyase	aspA
x	x	x	acetyltransferase	BC0849
x	x	x	ornithine--oxo-acid transaminase	rocD
x	x	x	2-oxoglutarate dehydrogenasesubunit E1	sucA
x	x	x	branched-chain aminoacid aminotransferase	BC1396
x	x	x	acetolactate synthasesmall subunit	ilvH
x	x	x	ketol-acid reductoisomerase	BC1399
x	x		2-isopropylmalate synthase	BC1400
x	x		3-isopropylmalate dehydrogenase	BC1401
x	x		3-isopropylmalate dehydrataselarge subunit	BC1402
x	x		asparagine synthetaseAsnA	BC1746
x	x	x	branched-chain aminoacid aminotransferase	BC1776
x	x	x	acetolactate synthase small subunit	BC1777
x	x	x	alcohol dehydrogenase	BC2220
x	x	x	citrate synthase3	BC2285
x			3-hydroxyisobutyrate dehydrogenase	BC2289
x			methylmalonate-semialdehyde dehydrogenase	acylating
x	x		metal-dependent hydrolase	BC2758
x	x	x	dihydrolipoamide dehydrogenase	acoL
x	x	x	acetoin dehydrogenase E1 component beta-subunit	BC2778
x	x	x	malate:quinone oxidoreductase	BC2959
x	x	x	aldehyde dehydrogenase	BC3555
x	x	x	aconitate hydratase	BC3616
x	x		imidazolonepropionase	BC3650
x	x		urocanate hydratase	BC3651
x	x		histidine ammonia-lyase	hutH
x	x	x	succinyl-CoA synthetasesubunit alpha	BC3833
x		x	succinyl-CoA synthetasesubunit beta	sucC
x		x	acetyl-CoA acetyltransferase	BC4023
x		x	glycine dehydrogenasesubunit 2	BC4224

x	x	x	glycine dehydrogenasesubunit 1	BC4225
x	x	x	glycine cleavage system aminomethyltransferase T	gcvT
x	x	x	bifunctional acetaldehyde-CoA/alcoholdehydrogenase	BC4365
x	x	x	succinate dehydrogenaseiron-sulfur subunit	sdhB
x	x	x	succinate dehydrogenaseflavoprotein subunit	sdhA
x	x	x	malate dehydrogenase	BC4592
x	x	x	isocitrate dehydrogenase	BC4593
x	x	x	citrate synthase	BC4594
x	x	x	acetyl-coenzyme Asynthetase	BC4645
x	x	x	phosphoenolpyruvate carboxykinase	BC4762
x	x	x	L-lactate dehydrogenase	ldh
x	x	x	L-lactate dehydrogenase	ldh
x		x	acetyl-CoA acetyltransferase	BC5003
x	x		enoyl-CoA hydratase	BC5004
x	x	x	Prolyne dehydrogenase	BC5006
x		x	acetyl-CoA acetyltransferase	BC5344

Supplementary Table 6. Specialization of biofilm and floating cells in distinct but coordinated metabolic activities. Genes belonging to cluster 2.

GeneIDs	Planktonic(blue)/Biofilm(red)	DAVID functional annotation
BC0049	red	SspF protein
BC0069	red	stage II sporulation protein E
BC0169	red	spore germination protein GerD
BC0170	blue	#N/A
BC0263	red	#N/A
BC0520	blue	#N/A
BC0603	red	hypothetical protein
BC0875	red	small acid-soluble spore protein
BC0877	red	hypothetical protein
BC1012	blue	#N/A
BC1225	blue	#N/A
BC1500	red	hypothetical protein
BC1501	red	hypothetical protein
BC1502	red	hypothetical protein
BC1509	red	stage IV sporulation protein A
BC1520	red	#N/A
BC1984	red	small acid-soluble spore protein
BC1994	blue	#N/A
BC2010	red	#N/A
BC2050	red	#N/A
BC2095	red	hypothetical protein
BC2536	red	cell wall hydrolase cwlJ
BC3106	red	small acid-soluble spore protein
BC3605	red	#N/A
BC3770	red	spore coat protein E
BC3783	red	#N/A
BC3800	red	dipicolinate synthase subunit B
BC3801	red	dipicolinate synthase subunit A
BC3802	red	hypothetical protein
BC3902	red	hypothetical protein
BC3905	red	sporulation sigma-E factor processing peptidase
BC3922	red	prespore specific transcriptional activator rsfA
BC4067	red	stage V sporulation protein AD
BC4073	red	anti-sigma F factor
BC4088	red	hypothetical protein
BC4186	red	stage III sporulation protein AH
BC4187	red	stage III sporulation protein AG
BC4188	red	stage III sporulation protein AF
BC4191	red	stage III sporulation protein AC
BC4192	red	stage III sporulation protein SpoAB
BC4193	red	#N/A

BC4194	red	hypothetical protein
BC4288	blue	membrane-attached cytochrome c550
BC4440	red	#N/A
BC4466	red	CotS-related protein
BC4467	red	stage VI sporulation protein D
BC4495	red	germination protein germ
BC4563	red	small acid-soluble spore protein SspI
BC4577	red	hypothetical protein
BC4606	red	hypothetical protein
BC4640	red	hypothetical protein
BC4641	red	hypothetical protein
BC4642	blue	inorganic polyphosphate/ATP-NAD kinase
BC4660	blue	#N/A
BC4662	blue	#N/A
BC4899	red	hypothetical protein
BC4923	blue	#N/A
BC4924	blue	#N/A
BC5145	red	hypothetical protein
BC5147	red	stage V sporulation protein AC
BC5148	red	stage V sporulation protein AD
BC5149	red	stage V sporulation protein AE
BC5282	red	stage III sporulation protein D
BC5283	red	stage II sporulation protein Q
BC5287	red	stage II sporulation protein D
BC5289	red	#N/A
BC5385	red	prespore specific transcriptional activator rsfA
BC5390	red	cell wall hydrolase cwlJ
BC5480	red	hypothetical protein
BC5391	red	hypothetical protein
BC4607	red	hypothetical protein

Supplementary Table 7. Specialization of biofilm and floating cells in distinct but coordinated metabolic activities. Genes belonging to cluster 3.

GeneIDs	Planktonic(blue)/Biofilm(red)	DAVID functional annotation
BC0114	blue	RNA polymerase factor sigma-70
BC0404	blue	methyl-accepting chemotaxis protein
BC0405	blue	#N/A
BC0422	blue	methyl-accepting chemotaxis protein
BC0559	blue	methyl-accepting chemotaxis protein
BC0576	blue	methyl-accepting chemotaxis protein
BC0678	blue	methyl-accepting chemotaxis protein
BC0679	red	#N/A
BC1625	blue	flagellar motor protein MotP
BC1626	blue	#N/A
BC1627	blue	chemotaxis protein CheY
BC1628	blue	chemotaxis protein CheA
BC1629	blue	flagellar motor switch protein
BC1630	blue	hypothetical protein
BC1636	blue	flagellar hook-associated protein FlgK
BC1637	blue	flagellar hook-associated protein FlgL
BC1638	blue	flagellar capping protein
BC1639	blue	flagellar protein fliS
BC1643	blue	flagellar hook-basal body protein FliE
BC1644	blue	flagellar MS-ring protein
BC1645	blue	flagellar motor switch protein G
BC1646	blue	flagellar assembly protein H
BC1647	blue	flagellum-specific ATP synthase
BC1651	blue	flagellar hook protein FlgE
BC1653	blue	hypothetical protein
BC1654	blue	chemotaxis protein CheV
BC1657	blue	flagellin
BC1658	blue	flagellin
BC1659	blue	flagellin
BC1660	blue	soluble lytic murein transglycosylase
BC1662	blue	flagellar motor switch protein FliM
BC1664	blue	flagellar motor switch protein fliN
BC1671	blue	flagellar basal body rod protein FlgG
BC1672	blue	metal-dependent hydrolase related to alanyl-tRNA synthetase
BC2006	blue	methyl-accepting chemotaxis protein
BC2766	blue	#N/A
BC3903	red	sporulation sigma factor SigG
BC3904	red	sporulation sigma factor SigE
BC4071	red	Sodium/proline symporter
BC4072	red	sporulation sigma factor SigF
BC4074	red	anti-sigma F factor antagonist
BC4512	blue	flagellar motor protein MotB

BC4513	blue	flagellar motor protein MotA
BC5009	blue	methyl-accepting chemotaxis protein
BC5034	blue	methyl-accepting chemotaxis protein
BC5065	blue	#N/A
BC5143	blue	#N/A
BC1631	blue	hypothetical protein
BC1640	blue	hypothetical protein
BC1648	blue	cytoplasmic protein
BC1652	blue	hypothetical protein
BC5035	red	#N/A
BC5066	blue	endonuclease/exonuclease/phosphatase family protein

Supplementary Table 8. Primers used to obtain mutants in *eps1* and *eps2*.

Mutation	Name	Sequence
ΔBC_1583 – 1591	Forward.up. BamHI	AAAAGGATCCGGGATGTTGCATAAGTCGAAC
	Reverse.up	ATTGGCATTAATCCAGCAAGGCCACGAGCGATTAAAC CATTC
	Forward.down	CCTTGCTGGATTAATGCCAAT
	Reverse.down.NcoI	AAAACCATGGCTCTTT TTCATTACCTATATCCCACTA
ΔBC_5279 - 5274	Forward.up. BamHI	AAAAGGATCCTTTGAAAGAACTAAGGCTGACG
	Reverse.up	TGGTTTTCTTTTCTTTCTCCCGAACATAT T
	Forward.down	AAACCATTACGACAATTAATTACACATCAGATGAGTCA AGA GAGTATTAGGAAAACC
	Reverse.do wn.NcoI	AAAACCATGGATGCAAAGGTAACGTGATTTCATT
